# Exploring the alternative virulence determinants PB2 S155N and PA S49Y/D347G that promote mammalian adaptation of the H9N2 avian influenza virus in mice

**DOI:** 10.1186/s13567-023-01221-6

**Published:** 2023-10-19

**Authors:** Yanna Guo, Xuebing Bai, Zhiyuan Liu, Bing Liang, Yiqing Zheng, Samar Dankar, Jihui Ping

**Affiliations:** 1 MOE International Joint Collaborative Research Laboratory for Animal Health and Food Safety & Jiangsu Engineering Research Center of Animal Immunology, College of Veterinary Medicine, Nanjing Agricultural University, Nanjing, 210095 China; 2 Department of Biochemistry, Microbiology and Immunology, Faculty of Medicine, University of Ottawa, Ottawa, ONK1V 8M5 Canada

**Keywords:** H9N2, cross-host infection, mouse adaptation, pathogenicity

## Abstract

The occurrence of human infections caused by avian H9N2 influenza viruses has raised concerns regarding the potential for human epidemics and pandemics. The molecular basis of viral adaptation to a new host needs to be further studied. Here, the bases of nucleotides 627 and 701 of PB2 were changed according to the uncoverable purine-to-pyrimidine transversion to block the development of PB2 627K and 701N mutations during serial passaging in mice. The purpose of this experiment was to identify key adaptive mutations in polymerase and NP genes that were obscured by the widely known host range determinants PB2 627K and 701N. Mouse-adapted H9N2 variants were obtained via twelve serial lung-to-lung passages. Sequence analysis showed that the mouse-adapted viruses acquired several mutations within the seven gene segments (PB2, PB1, PA, NP, HA, NA, and NS). One variant isolate with the highest polymerase activity possessed three substitutions, PB2 S155N, PA S49Y and D347G, which contributed to the highly virulent and mouse-adaptative phenotype. Further studies demonstrated that these three mutations resulted in increased polymerase activity, viral transcription and replication in mammalian cells, severe interstitial pneumonia, excessive inflammatory cellular infiltration and increased growth rates in mice. Our results suggest that the substitution of these three amino acid mutations may be an alternative strategy for H9N2 avian influenza viruses to adapt to mammalian hosts. The continued surveillance of zoonotic H9N2 influenza viruses should also include these mammalian adaptation markers as part of our pandemic preparedness efforts.

**Supplementary Information:**

The online version contains supplementary material available at 10.1186/s13567-023-01221-6.

## Introduction

The H9N2 subtype of avian influenza virus is the predominant virus in the live poultry market in China. It is capable of spreading quickly among poultry, resulting in decreased egg production and increased poultry mortality, leading to considerable economic damage to the farming industry [1, 2]. The H9N2 virus prevails in multiple avian species, including chicken, duck, quail, and pigeon, and can also cross host barriers to infect humans. The first human case of the H9N2 subtype of influenza virus was reported in 1988 in South China, and numerous incidents involving this type of virus have been reported in succession [3–7]. Because the majority of H9N2 cases are not severe and the viruses are not transmissible between humans, they are usually overlooked [5, 8], which has allowed the viruses to continue to evolve and spread. However, it is worth noting that a few H9N2 viruses are increasingly adapting to mammals [2]. Research has indicated that the H9N2 strain isolated prior to 2000 was not able to replicate in mice, resulting in no visible disease symptoms. However, the H9N2 virus isolated from 2000 to 2002 was found to cause a loss of approximately 20% of the initial body weight of mice [9]. Two studies have demonstrated that some H9N2 viruses isolated from 2007 to 2009 were highly lethal in mice [10, 11]. In addition, numerous human infections with avian influenza virus revealed that the H9N2 influenza virus is the gene donor for H5N1 [12], H5N6 [13], H7N9 [14], H10N8 [15] and H10N3 [16], which infect humans. This information suggests that the threat of the H9N2 virus to public health is indeed increasing.

One of the most significant factors limiting infection by avian influenza viruses across species is their poor polymerase activity in mammalian cells [17]. However, viruses have developed the capacity to circumvent this barrier by acquiring adaptive mutations in the polymerase and NP proteins [18]. Determining adaptations using the successive passages of avian influenza viruses in mammals (e.g., mice and ferrets) is an ideal means of studying the cross-species transmission of influenza viruses. Experiments involving influenza virus infection in a mouse model are highly beneficial for understanding mammalian disease and adaptation, while data from this model provide further insight into the molecular mechanisms required for mammalian adaptation. Systematic analysis of the literature related to the evolution of adaptation in new hosts revealed that most avian influenza viruses rapidly adapt and cause high pathogenicity in new hosts by acquiring the PB2 627K or 701N mutation immediately after only 2–3 consecutive passages [19–21]. However, the presence of the 627K and 701N mutations often masks the function of other mammalian adaptive mutations or affects the evolution of other genes and residues while greatly increasing the difficulty of monitoring new strains that are highly pathogenic in mammals and humans. How can the impact of the rapid acquisition of the PB2 627K and 701N mutations by influenza viruses be reduced during mammalian transmission? Are mutations in the polymerase and other residues of the NP gene of avian influenza viruses able to compensate for the function of the PB2 627 and 701 mutations? No in-depth study has yet been conducted.

The ribonucleoprotein complex is a critical factor in the determination of the influenza virus host range and adaptation to new species, making it a central subject in genetic research. In this study, 627E and 701D in the PB2 gene of the H9N2 subtype avian influenza virus were mutated to serine (S) and leucine (L) by using site-directed mutagenesis, respectively, to achieve a purine-to-pyrimidine transversion of all three or two nucleotides encoding amino acids at the indicated residues. This ensured that PB2 627E and 701D would not acquire the 627K and 701N mutations during passages in mice, which allowed us to screen for virulence-determining molecular markers in RNPs that were masked by the acquisition of 627K and 701N.

## Materials and methods

### Cells and viruses

Human embryonic kidney (293T), chicken fibroblast (DF-1), human pulmonary adenocarcinoma (A549) and Madin-Darby canine kidney (MDCK) cells, obtained from ATCC (Manassas, VA, USA), were cultured in Dulbecco’s modified Eagle’s medium (DMEM) (HyClone, South Logan, UT, USA) with 10% foetal bovine serum (FBS) and incubated at 37 °C with 5% CO2. The wild-type vaccine-like H9N2 virus strain A/chicken/Jiangsu/875/2018 (H9N2, abbreviated as JS/875) containing PB2 627E and 701D was isolated in Jiangsu Province in 2018 during the routine surveillance of a live poultry market and purified through three rounds of plaque purification. The virus was propagated in the allantoic cavities of 9-day-old SPF chicken eggs for 48 h at 37 °C, and the viral allantoic fluid was collected and stored at −80 °C until use.

### Construction of plasmids and generation of recombinant viruses

The total RNA of JS/875 was extracted by using TRIzol Reagent (Invitrogen) according to the manufacturer’s instructions. The full-length sequences of the eight gene segments were amplified with gene-specific primers and then cloned and inserted into the pHH21 vector. Mutant plasmids were generated by site-directed mutagenesis. All plasmids were sequenced to verify the presence of the introduced mutation and the absence of unwanted mutations. Virus rescue was performed as previously described [22]. Briefly, 200 ng of each of the eight vRNA transcriptional plasmids PB2, PB1, PA, NP, HA, NA, M, and NS (or mutant plasmid) along with 1 µg of each of the four protein expression plasmids were mixed with Opti-MEM. The transfection reagent Lipofectamine 2000 (Invitrogen) was added to another tube containing Opti-MEM at a dose of 1 µg plasmid with 2 µL Lipofectamine 2000 and mixed gently. After that, the two reagents were mixed well and incubated at room temperature for 25 min and then added to an 80% confluent monolayer of 293T cells in a 35 mm dish. Twenty hours later, the supernatant was discarded and replaced with fresh Opti-MEM containing 1 µL of tosylsulfonyl phenylalanyl chloromethyl ketone (TPCK)-trypsin (1 µg/mL). Forty-eight hours post transfection, the supernatants were collected and inoculated into 10-day-old specific-pathogen-free SPF embryonated chicken eggs for virus propagation. Forty-eight hours post infection, viral allantoic fluid was harvested, and virus titres were detected by plaque assay in MDCK cells. All eight genes were fully sequenced to ensure the absence of unwanted mutations.

### Minigenome assays

To determine the relative polymerase activity of different H9N2 variants, the PB1, NP and wild-type and mutant PB2 and PA genes were introduced into the pCAGGS protein expression vector. Human 293T and avian DF-1 cells (80% confluent monolayer cells in 24-well plates) were transfected with four protein expression plasmids for wild-type or mutant PB2, PB1, wild-type or mutant PA and NP proteins (150 ng of each protein), together with 150 ng of reporter plasmid for the expression of a virus-like RNA encoding the firefly luciferase gene under the control of the human or chicken polymerase Ι promoter and 50 ng of the internal control plasmid encoding Renilla luciferase. Cells were incubated for 48 h at 33 °C or 37 °C for 293T cells and for 48 h at 37 °C or 39 °C for DF-1 cells. The cells were lysed, and the relative luciferase activity was measured by using a dual-luciferase reporter assay kit (Promega, Madison, WI, USA) according to the manufacturer’s instructions. Data shown are the mean ± SD for the results of three independent experiments.

### Lung-to-lung serial passage in mice

A recombinant H9N2 mutant virus, rJS/875-PB2 627S+701L, was generated by reverse genetics. Three specific pathogen-free female BALB/c mice were used to conduct an independent series of lung-to-lung passages, as described previously [23]. Briefly, mice were anaesthetized with isoflurane and inoculated intranasally with 50 µL of rJS/875-PB2 627S+701L virus stock. Three days post-inoculation, the mice were euthanized, and the lungs were collected and homogenized. Then, the viral supernatant was used to inoculate the next female BALB/c mice. This procedure was repeated for 12 passages, and we obtained the highly virulent rJS/875-PB2 627S+701L-MA. rJS/875-PB2 627S+701L-MA was purified and propagated in MDCK cells and stored at −80 ℃.

### Plaque assays for virus purification

For virus purification, the infected mouse lung tissues were homogenized, and the supernatant was obtained by centrifugation for plaque assays to isolate several virulent mouse-adapted viruses. Virus supernatants were serially diluted tenfold in DMEM, added to MDCK cells in six-well plates, and then incubated at 37 ℃ for 1 h. After 1 h of infection, the cells were washed with PBS and the medium was replaced with a mixture of 2% agarose medium and 2× DMEM containing 0.3% bovine serum albumin (BSA), 1 µg/mL TPCK-trypsin and 10 µL/mL HEPES. The agarose was left to solidify at room temperature, and then the plates were placed in an inverted position at 37 ℃ for 48 h. Forty-eight hours later, twelve single-plaque colonies were picked and resuspended in medium. Then, the next round of plaque purification was performed, and this procedure was repeated three times. Finally, the last viral suspension was propagated in 11-day-old embryonated chicken eggs. Forty-eight hours later, viruses were harvested, and virus purification was completed.

### Virus titration

Virus titres were also determined by plaque assay as described previously. Briefly, 400 µL of serial tenfold dilutions of virus were inoculated into MDCK cells in a six-well plate. After 1 h of incubation, the cells were washed twice in PBS, and the mixture was replaced with 2% agarose medium and 2× DMEM containing 0.3% bovine serum albumin (BSA), 1 µg/mL TPCK-trypsin and 10 µL/mL HEPES. Forty-eight hours later, the cells were fixed with 10% formaldehyde and stained with 0.5% crystal violet solution. The number of plaques was counted, and the virus titre was calculated as PFU/mL.

### Genomic sequencing of the mouse-adapted viruses

Viral RNA of the mouse-adapted viruses was extracted using TRIzol Reagent (Invitrogen) according to the manufacturer’s instructions and used as a template for genomic amplification. RT‒PCR was performed by using influenza A virus-specific primers as described previously [24]. The 50 µL PCR contained 25 µL of 2× Phanta Max Buffer, 1 µL of upstream primers, 1 µL of downstream primers, 1 µL of dNTP mix, 1 µL of Phanta Max Super-Fidelity DNA Polymerase and 1 µL of viral RNA. The genome was sequenced by Sangon Biotech, and adaptive mutations obtained from serial passage were identified by comparing the JS/875-PB2 627S+701L-MA sequence with the sequence of the parent virus JS/875-PB2 627S+701L.

### Mouse experiments

Specific pathogen-free 6-week-old female BALB/c mice were supplied by Xipuer-bikai Experimental Animal Company (Shanghai, China) and used in this study. Five mice per group were inoculated intranasally with the indicated viruses. Then, the mice were monitored daily to record body weight changes and mortality for 14 days post-infection. Mice that lost more than 25% of their initial body weight were euthanized. To determine the replication ability of viruses in mouse tissue organs, groups of 3 mice were inoculated intranasally with the indicated viruses and euthanized on day 3 post-infection. Nasal turbinates and lungs were collected for virus titration by plaque assay using MDCK cells. For pathological examination, virus-infected lungs on day 3 post-infection were fixed in 10% formaldehyde for 48 h. The samples were then placed in paraffin, and thin slices of 4 μm were cut and stained with haematoxylin and eosin (H&E) for examination under light microscopy (Nikon, Japan). The data shown are the mean ± SD of three independent experiments.

### Viral replication assay

Triplicate wells of confluent monolayers of A549 and MDCK cells were infected with different viruses at a multiplicity of infection (MOI) of 0.05 and 0.001, respectively, and incubated for 1 h at 37 °C. One hour post-infection, cells were further incubated in DMEM containing 0.3% bovine serum albumin (BSA), 1 µg/mL TPCK-trypsin and 10 µL/mL HEPES at 37 °C. The supernatant was collected at 12, 24, and 48 h post-infection. Virus titres in the culture supernatants were determined by plaque assay using MDCK cells.

### Quantitation of viral vRNA and mRNA levels in A549 cells and selected cytokines and chemokines in infected mouse lungs

The relative expression levels of viral mRNA and vRNA were determined in different virus-infected A549 cells at an MOI of 2. Total RNA was extracted from the infected A549 cells at 8 hours post infection by using TRIzol reagent according to the manufacturer’s instructions. For the detection of viral mRNA and vRNA, oligo dT and NP vRNA primers (5′-AGCAAAAGCAGGGTAGATAATCACT-3′) were used to generate cDNAs with 1 µg of total RNA per sample according to the Hiscript II 1st Strand cDNA Synthesis Kit (Vazyme). To determine the relative expression levels of interferon beta (IFN-β), interleukin-6 (IL-6) and interleukin-1 beta (IL-1β) in the infected mouse lung tissues, total RNA was extracted from the different virus-infected lung tissues on day 3 post-inoculation. Random and oligo dT were used to generate cDNAs from 1 µg of total RNA per sample. qPCR was performed in accordance with the instructions provided by the manufacturer of the AceQ qPCR SYBR Green Master Mix (Vazyme) on a LightCycler® 96 system. The reaction mixture contained 10 µL of 2× SYBR green PCR master mix, 7.2 µL of nuclease-free water, 0.4 µL of each primer and 2 µL of cDNA template, and the cycling conditions were as follows: 95 °C for 5 min, followed by 40 cycles of 95 °C for 10 s and 60 °C for 30 s. The expression values of each gene relative to GAPDH were calculated using the 2^−ΔΔCT^ method. The primers used in this study are provided in Additional file 1.

## Results

### Recombinant H9N2 virus containing PB2 627S and 701L has low polymerase activity in 293T cells and leads to moderate mortality in mice

**Figure 1:**
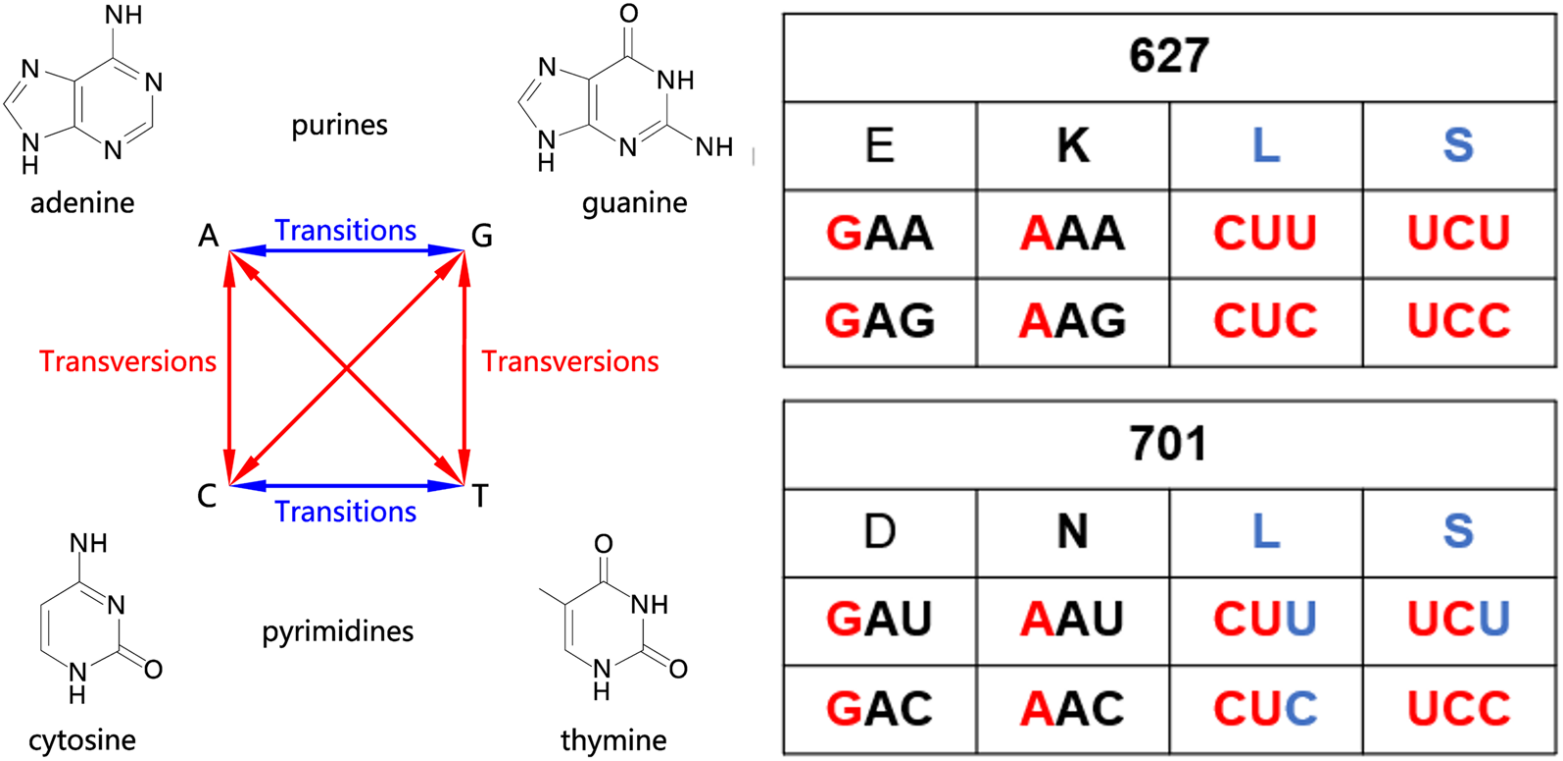
**Base transition and transversion of the PB2 627 and 701 residues.**

**Figure 2:**
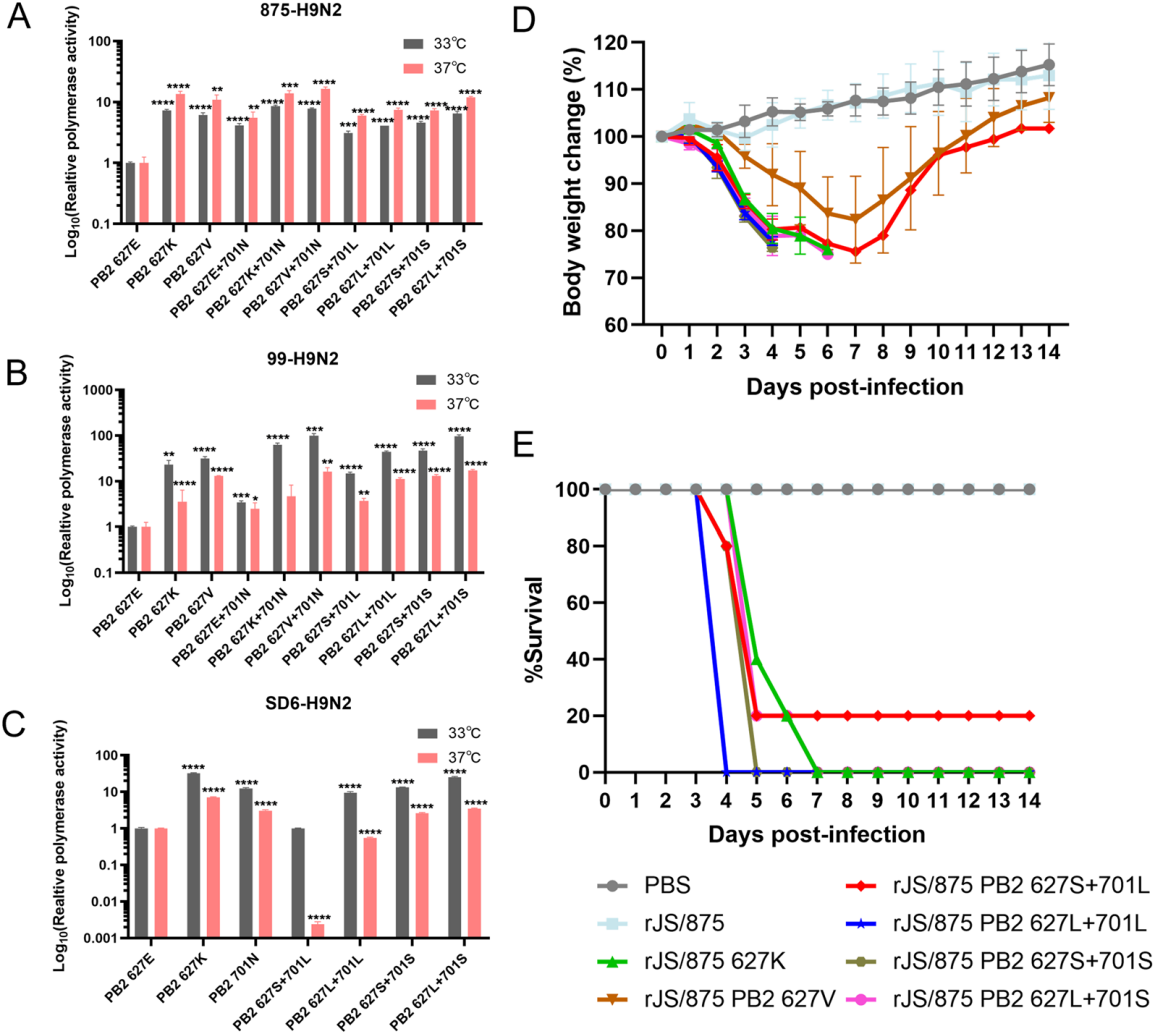
**Viral polymerase activities of different PB2 mutants and their virus pathogenicity in mice.** The polymerase activity of RNP complexes reconstituted from the PB2, PB1, PA and NP plasmids of the A/chicken/Jiangsu/875/2018 (**A**), A/chicken/Jiangsu/99/2017 (**B**) and A/chicken/Shandong/6/1996 (**C**) viruses and their single or double mutants were assessed by a dual luciferase reporter assay in three independent experiments. *P* values were calculated by using the Student’s *t* test, *P < 0.05, **P < 0.01, ***P < 0.001, ****P < 0.0001. Six-week-old female BALB/c mice (*n* = 5/group) were inoculated intranasally with 4 × 10^5^ PFU of rJS/875 WT or its PB2 mutant viruses or an equal volume of PBS as the mock inoculation. Body weight changes (**D**) and survival rate (**E**) were recorded as the percentage of the weight on the day of inoculation (day 0). Mice that lost more than 25% of their original body weight were euthanized. The data presented are the mean ± SD of three independent experiments.

The PB2 E627K and D701N mutations are crucial molecular indicators for avian influenza virus that are required to transmit across species and infect mammals. Base mutation is a driving force in the process of gene evolution, and the probability of base transition is obviously greater than that of transversion [25]. E627K and D701N mutations require only one base transition, which is extremely easily achieved during evolution. To limit the likelihood of occurrence of the E627K and D701N mutations in our mouse adaptation study, the sites 627E and 701D of the vaccine-like H9N2 virus JS/875 were mutated to 627L/S and 701L/S, respectively. This approach was used to ensure that the likelihood of 627L/S and 701L/S mutating to 627K and 701N was extremely low since it required five of six bases to undergo transversions (Figure 1). Our only aim here was to reduce the likelihood of reverting back to 627K and 701N during viral evolution in order to identify other important mammalian adaptive molecular markers that were masked by the 627K and 701N mutations in the polymerase and NP genes. The JS/875 PB2-627S+701L mutant demonstrated the least polymerase activity among the tested mutations at both 33 and 37 °C (Figure 2A). Two other H9N2 vaccine strains, A/chicken/Jiangsu/99/2017 (H9N2, 99-H9N2) and A/chicken/Shandong/6/1996 (H9N2, SD6-H9N2), also yielded the same results (Figures 2B, C). Mouse pathogenicity studies have shown that the rJS/875-PB2 627S+701L virus has relatively moderate virulence in mice infected by inoculation with a dose of 4 × 10^5^ PFU/mouse (Figures 2D, E). Therefore, we selected the rJS/875-PB2 627S+701L virus for the subsequent serial lung-to-lung passage in mice to screen for potential mouse-adapted molecular markers in the polymerase and NP genes.

### Generation of mouse-adapted (MA) H9N2 viruses and sequencing

To imitate the adaptation process of H9N2 AIV in mammals, the rJS/875-PB2 627S+701L strain was serially passaged through mouse lungs by intranasal inoculations. After twelve successive passages, mouse-adapted variants were obtained from three mice. To acquire a single, purified mouse-adapted H9N2 virus, a plaque assay for purification was performed on the twelve passaged mouse-infected lungs using MDCK cells and repeated three times. Twelve plaques were randomly chosen from the plaque-purified viruses from the three mouse-adapted lungs for propagation. The full-length sequences of all segments of the twelve purified viruses were analysed and compared to that of the parental strain rJS/875-PB2 627S+701L, and the mouse-adapted mutations identified are shown in Table 1. MA#1 to MA#5 were isolated from the first mouse. MA#6 to MA#9 were isolated from the second mouse. MA#10 to MA#12 were isolated from the third mouse. Sequencing results showed that two purified viruses, MA#4 and MA#8, shared the same sequences as MA#2 and MA#6 in the polymerase and NP genes, respectively. The remaining ten mouse-adapted viruses possessed several mutations within seven gene segments when compared with the parental virus rJS/875-PB2 627S+701L, including two in the PB2 gene (S155N, K526R), two in the PB1 gene (V418I, L695I), seven in the PA gene (S49Y, T97I, I178M, D347G, M374T, V450A, E684G), one in the NP gene (V183I), nine in the HA gene, seven in the NA gene, and one in the NS gene (Table 1). All subsequent experiments were performed with the wild-type strain rJS/875, which contains PB2 627E and is present in nature.

**Table 1:** **Amino acid sequence differences between the parental wild-type and mouse-adapted viruses**

Gene	Positions (aa)		Parental virus	Mouse adapted viruses (MA#)											
				1	2	3	4	5	6	7	8	9	10	11	12
PB2	155		S												N
	526		K			R									
PB1	418		V							I					
	695		L	I	I		I								
PA	49		S										Y	Y	Y
	97		T	I	I	I	I	I	I	I	I	I			
	178		I	M		M		M							
	347		D										G	G	G
	374		M	T		T		T							
	450		V					A							
	684		E									G			
NP	183		V										I		
HA	138		A						T						
	155		T										I		
	182		I							L					
	187		T	P	P	P	P	P							
	205		A				E		E	E	E	E			
	210		N												D
	218		G	E	E	E		E							
	221		P										A	A	
	463		G												E
NA	111		I						V	V	V	V			
	143		N	S											
	150		T					I							
	189		I				T								
	251		V										I		
	343		A	T											
	433		V		A										
NS1	176		N				I								

### Mouse-adapted H9N2 variants increase viral polymerase activity and pathogenicity in mice

**Figure 3:**
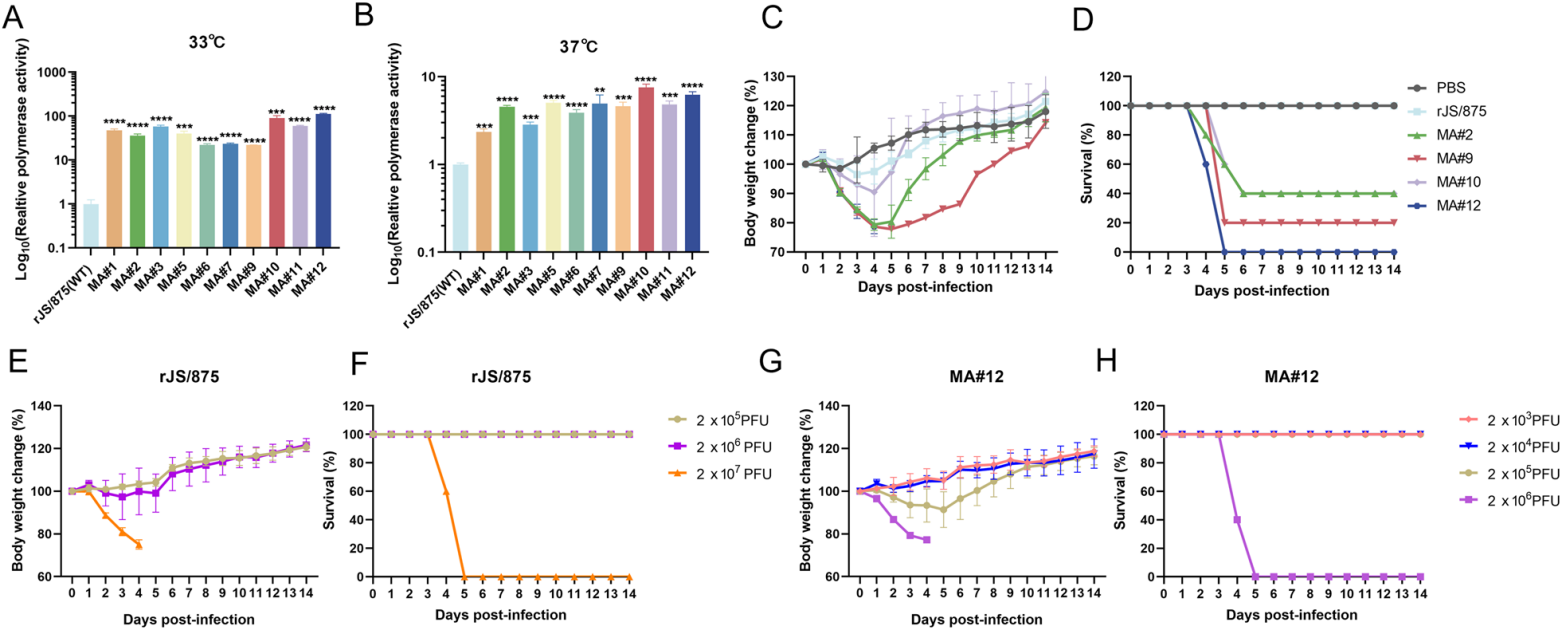
**Polymerase activities and viral pathogenicity of different mouse-adapted variants.** The polymerase activities of different mouse-adapted viruses were determined by using the minigenome assay in 293T cells at 33 °C (**A**) and 37 °C (**B**). The data presented are the mean ± SD of three independent experiments. *P* values were calculated by using Student’s *t* test, *P < 0.05, **P < 0.01, ***P < 0.001, ****P < 0.0001. Six-week-old female BALB/c mice (*n* = 5/group) were inoculated intranasally with 50 µL containing 2 × 10^6^ PFU of rJS/875 WT or the mouse-adapted viruses, and the PBS-infected group was used as the mock inoculation. **C** Body weight changes and **D** survival rate were monitored as the percentage of the weight on the day of inoculation (day 0). The MLD_50_ values of rJS/875 and MA#12 were also determined by inoculating groups of five 6-week-old female BALB/c mice with tenfold serial dilutions of different viruses in a 50 µL volume (2 × 10^5^–2 × 10^7^ PFU of rJS/875 or 2 × 10^3^–2 × 10^6^ PFU of MA#12). The body weight changes (**E**, **G**) and survival rate (**F**, **H**) of mice given these two viruses were assessed by measuring weight changes over a 14-day period and are depicted as a percentage of the animal’s weight on the day of inoculation (day 0). Mice that lost more than 25% of their original body weight were euthanized.

The mini-genome assay showed that all mouse-adapted strains demonstrated increased polymerase activity compared to the wild-type strain rJS/875, ranging from 22.06 to 112.75 times greater at 33 °C, with MA#9 exhibiting the least relative polymerase activity and MA#12 displaying the highest relative polymerase activity. At 37 °C, MA#1 had 2.34 times greater polymerase activity than rJS/875, while MA#10 exhibited 7.55 times greater polymerase activity than rJS/875; meanwhile, the remaining mouse-adapted strains demonstrated levels of polymerase activity lying between the two relative values (Figures 3A, B). Four mouse-adapted viruses were chosen and rescued by reverse genetics (all viruses possess PB2 627E) for mouse pathogenicity studies. These included the MA#10 and MA#12 strains isolated from the third mouse (MA#10 to MA#12) with the highest polymerase activity in 293T cells, which were most likely to promote virus virulence. Another two mouse-adapted mutant viruses with relatively moderate polymerase activity, MA#2 and MA9, were selected from the first (MA#1 to MA#5) and second mice (MA#6 to MA#9), respectively, as controls to verify the relationship between polymerase activity and pathogenicity. The results showed that all of the tested MA strains were more virulent than the rJS/875 virus. MA#12 was the most virulent, and the lethality rate reached 100% on the fifth day after infection (Figures 3C, D). The 50% mouse lethal dose (MLD_50_) of rJS875 and MA#12 was 6.3 × 10^6^ PFU and 6.3 × 10^5^ PFU, respectively (Figures 3E–H). Therefore, MA#12 exhibited the highest polymerase activity and pathogenicity in mice and was chosen for further analysis as the representative mammalian-adapted H9N2 isolate. Sequence analysis showed that the parental and mouse-adapted virus MA#12 exhibited five variations in amino acid composition. The PB2 protein underwent one amino acid mutation (S155N), and the polymerase acidic protein (PA) had two coding changes (S49Y and D347G). Moreover, there was one amino acid mutation in the HA (N210D, H3 number) and NA (G463E) proteins. Since the purpose of our experiment was to investigate the key mammalian adaptive molecular markers on the polymerase and NP genes, the following three sites on the polymerase genes were explored.

### The PB2 S155N and PA S49Y and D347G substitutions conferred increased JS/875 virulence in mice

**Figure 4:**
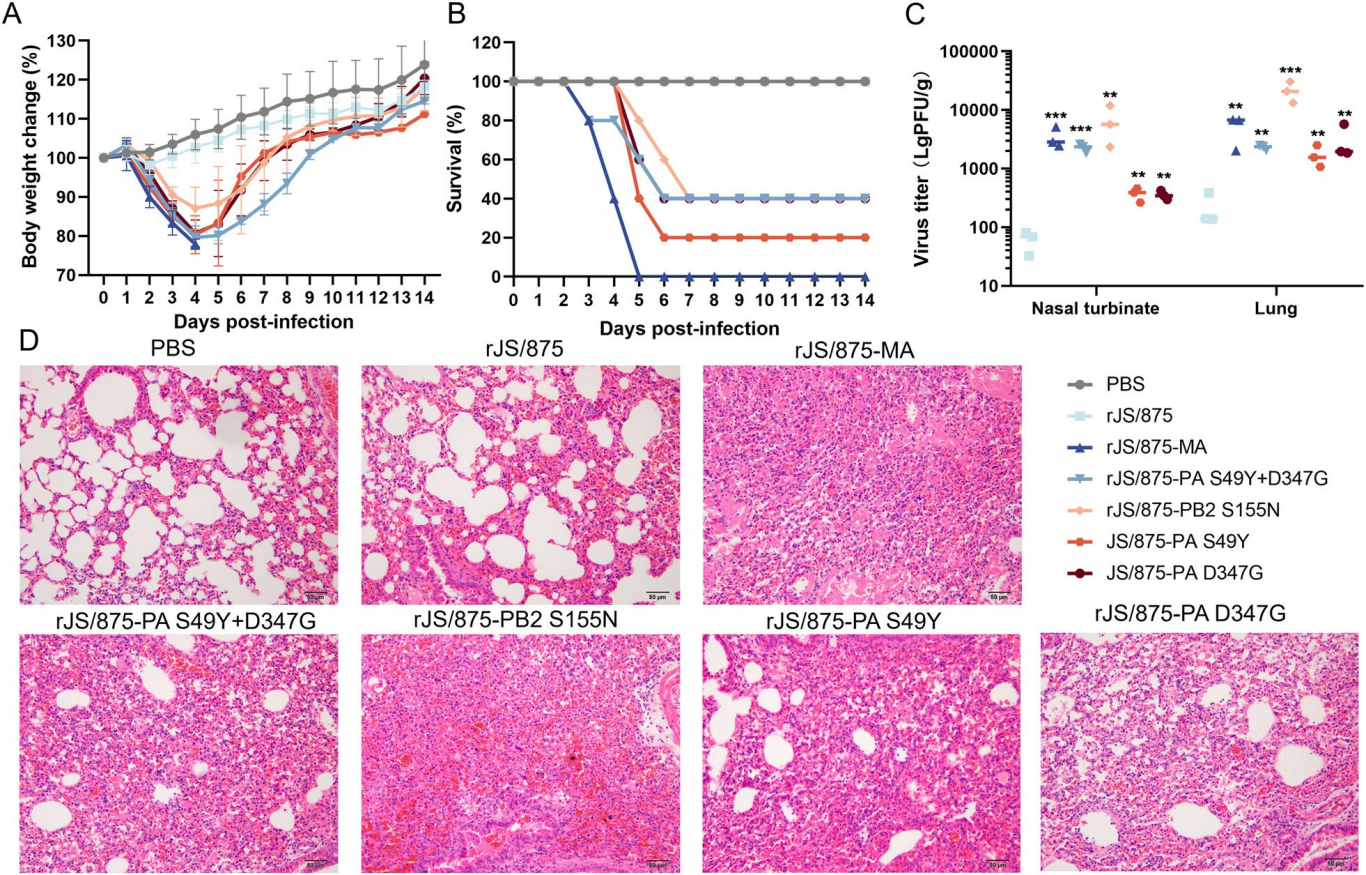
**Effect of the PB2 S155N and PA S49Y and D347G mutations on viral pathogenicity in mice.** Five 6-week-old female BALB/c mice were inoculated with 2 × 10^6^ PFU of the wild-type and PB2 and PA mutant viruses. Body weight loss (**A**) and mortality (**B**) were observed at 14 days after infection. Virus replication ability (**C**) in mouse turbinate and lung tissue and lung histopathology (**D**) on day 3 post-infection were determined. Mice that lost more than 25% of their original body weight were euthanized.

As the amino acid mutations occurred in two gene segments (PB2 and PA) of mouse-adapted virus, the recombinant virus JS/875 (rJS/875) and recombinant viruses containing the PB2 gene mutation (rJS/875-PB2 S155N), the PA mutated gene (rJS/875-PA S49Y+D347G) and the PA single point mutation (rJS/875-PA S49Y, rJS/875-PA D347G) and mouse-adapted virus (MA#12) rJS/875-MA were generated by reverse genetics using the JS/875 backbone. To further evaluate the contribution of each amino acid mutation to virulence in mice, a BALB/c mouse model was used. The body weight loss and survival rate of mice infected with each of these recombinant viruses were monitored for 14 days. For a given dose of 2 × 10^6^ PFU virus per mouse, all mice infected with rJS/875-MA showed signs of illness, such as reduced activity, huddling and horripilation. Mice in this virus-infected group showed the greatest loss of body weight (22.1%), and all mice died before day 5 post-infection. In contrast, no mortality was observed in the rJS/875-infected group at the same dose. Among the three point polymerase mutated viruses, the single amino acid substitution S155N in the PB2 gene moderately increased the pathogenicity of rJS/875 and exhibited moderate virulence with 60% mortality. The substitution of S49Y in PA relatively enhanced rJS/875 pathogenicity and resulted in a mortality rate of 80%. The PA-D347G mutation increased the pathogenicity of rJS/875, and mice infected with this mutant began to die on day 5 post-infection (Figures 4A, B).

To determine the replicative ability of different recombinant viruses in mouse tissues, the three infected mice in each group were euthanized and the nasal turbinate and lung samples were collected on day 3 post-infection for virus titration. As shown in Figure 4C, viruses containing all single mouse-adapted mutants produced higher virus titres in the lungs and turbinates than the wild-type virus rJS/875, and the recombinant virus rJS/875-PA S49Y replicated best in both nasal turbinates and lungs (Figure 4C).

H&E staining showed that the virus rJS/875 induced mild and limited interstitial pneumonia but also a thickening of alveolar walls and a small amount of inflammatory cell infiltration. Moderate interstitial pneumonia was observed in the lungs of mice infected with rJS/875-PA S49Y+D347G and rJS/875-PA D347G. However, rJS/875-MA, rJS/875-PB2 S155N and rJS/875-PA S49Y induced severe interstitial pneumonia, characterized by alveolar wall thickening, alveolar collapse and massive numbers of red blood cells, immune cells and inflammatory cells in the lung sections (Figure 4D).

In summary, these results indicate that the PB2 S155N and PA S49Y and D347G mutations selected upon mouse adaptation contribute to the virulence of rJS/875 in mammals.

### The PB2 S155N and PA S49Y and D347G mutations increased viral replication in mammalian cells

**Figure 5:**
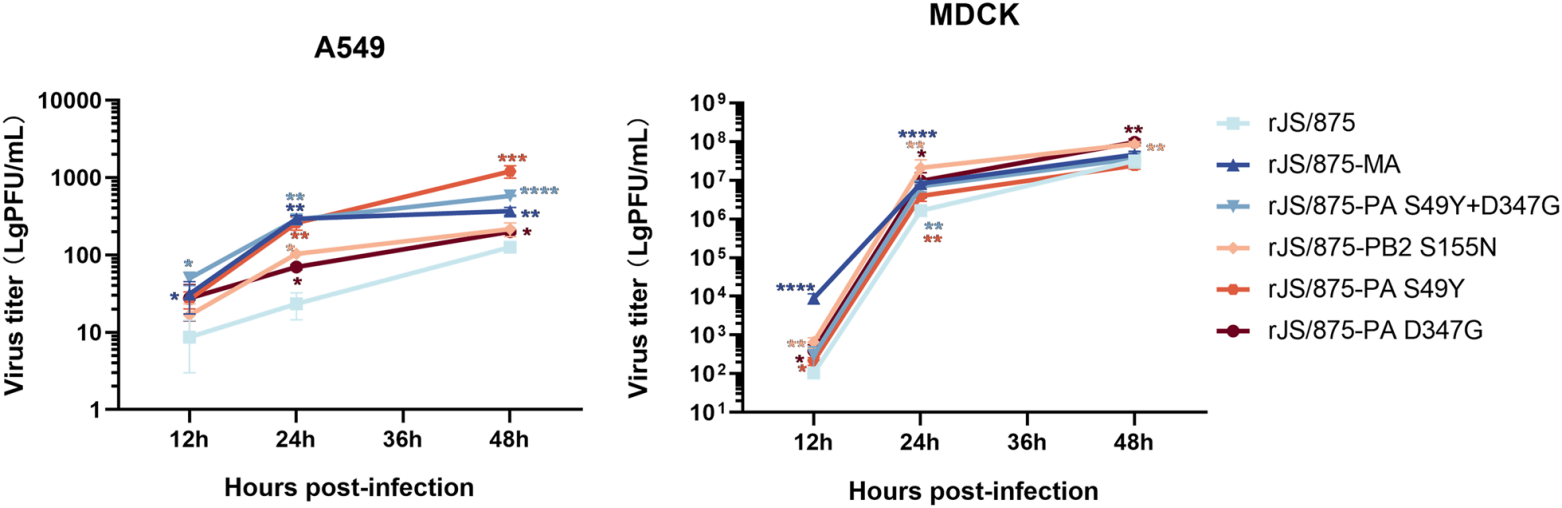
**Effect of the PB2 S155N and PA S49Y and D347G mutations on viral replication in mammalian cells.** A549 (**A**) or MDCK (**B**) cells were infected with wild-type and mutant viruses carrying these three mutant residues alone or simultaneously at an MOI of 0.01 or 0.001, respectively, and viral supernatants were collected at 12, 24 and 48 h post-infection to determine viral titres using a plaque assay. The data presented are the mean ± SD of three independent experiments. *P* values were calculated by using Student’s *t* test, *P < 0.05, **P < 0.01, ***P < 0.001, ****P < 0.0001.

To further validate the increase in replication in mammalian cells, the replication ability of recombinant viruses containing these key amino acid mutations was determined in MDCK and A549 cells at MOIs of 0.001 and 0.05, respectively. Supernatants were harvested at 12, 24, and 48 h post-infection, and virus titres were calculated. For the A549 cells (Figure 5A), the results showed that virus titres for rJS/875-MA were significantly higher than those for the wild-type strain rJS/875 at 12–48 h (*P* ≤ 0.05). Furthermore, the introduction of PB2 S155N into rJS/875 resulted in increased viral replication at 24 h post-infection. The mutation PA S49Y clearly enhanced virus replication ability at 24 and 48 h, and the replication difference compared to rJS/875 was large at 48 h (*P* ≤ 0.001). PA D347G slightly increased viral replication at 24–48 h (*P* ≤ 0.05). No additive effect was observed with the two PA mutations S49Y and D347G. For MDCK cells (Figure 5B), virus yields for rJS/875-MA were significantly higher than those for the wild-type strain rJS/875 at 12 and 24 h (*P* ≤ 0.0001). The substitution S155N in PB2 resulted in particularly increased replication from 12 to 48 h (*P* ≤ 0.01). Single S49Y and D347G mutations slightly increased virus replication at the early and middle stages of infection. Taken together, these data suggest that mammalian-adapted markers in PB2 (S155N) and PA (S49Y and D347G) can increase H9N2 virus replication in vitro.

### The PB2 S155N and PA S49Y and D347G mutations augment viral transcription and genomic replication

**Figure 6:**
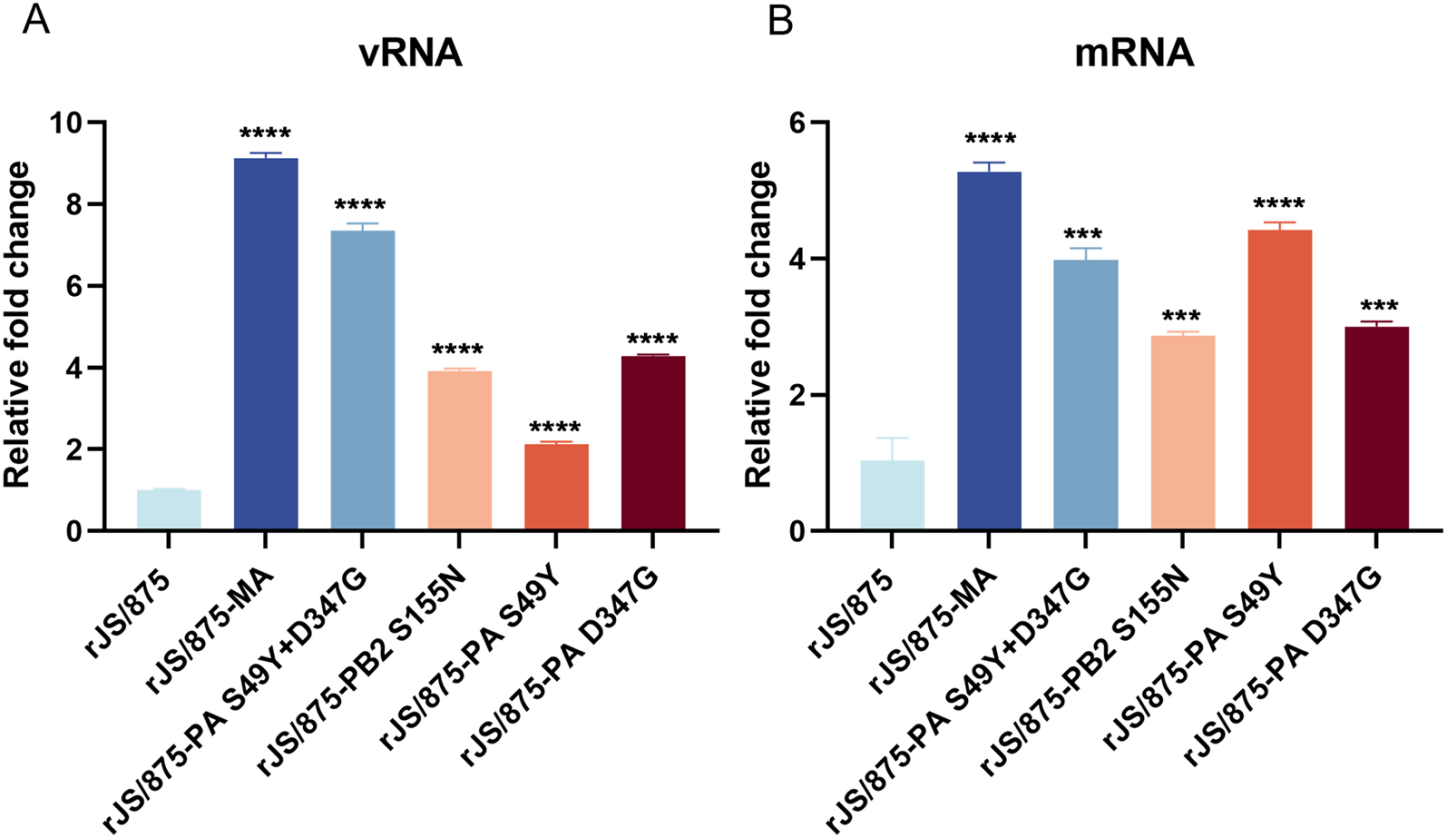
**The relative expression levels of viral vRNAs and mRNAs in A549 cells infected with recombinant H9N2 viruses carrying the PB2 S155N and PA S49Y and D347G mutations.** Monolayers of A549 cells were infected with different mutant viruses harbouring the PB2 S155N and PA S49Y and D347G mutations at an MOI of 2. The abundance of vRNAs and mRNAs encoding the NP gene of the indicated viruses is expressed as the fold-change in copy number compared with the wild-type group at 8 h post-infection. The data presented are the mean ± SD of three independent experiments. *P* values were calculated by using the Student’s *t* test, ***P < 0.001, ****P < 0.0001.

To investigate whether the introduction of the three key amino acid mutations in the PB2 and PA genes could affect viral vRNA and mRNA synthesis, virus-infected A549 cells were harvested at 8 h post-infection, and the synthesis of viral NP vRNA and mRNA was quantified (Figures 6A, B). The results showed that all mutant viruses with single or multiple mammal-adapted markers produced significantly higher levels of both viral NP vRNA and mRNA at 8 h post-infection compared to the wild-type H9N2 virus rJS/875 (*P* ≤ 0.001). The increase in rJS/875-MA was the most prominent, with a 9.13-fold increase in vRNA and a 5.28-fold increase in mRNA compared to rJS/875 (*P* ≤ 0.0001). These data demonstrated that the substitutions S155N in the PB2 gene and S49Y and D347G in the PA gene increased viral transcription and replication by increasing the synthesis of viral vRNA and mRNA in human A549 cells.

### The PB2 S155N and PA S49Y and D347G mutations contribute to cytokine induction in mouse lungs

**Figure 7:**
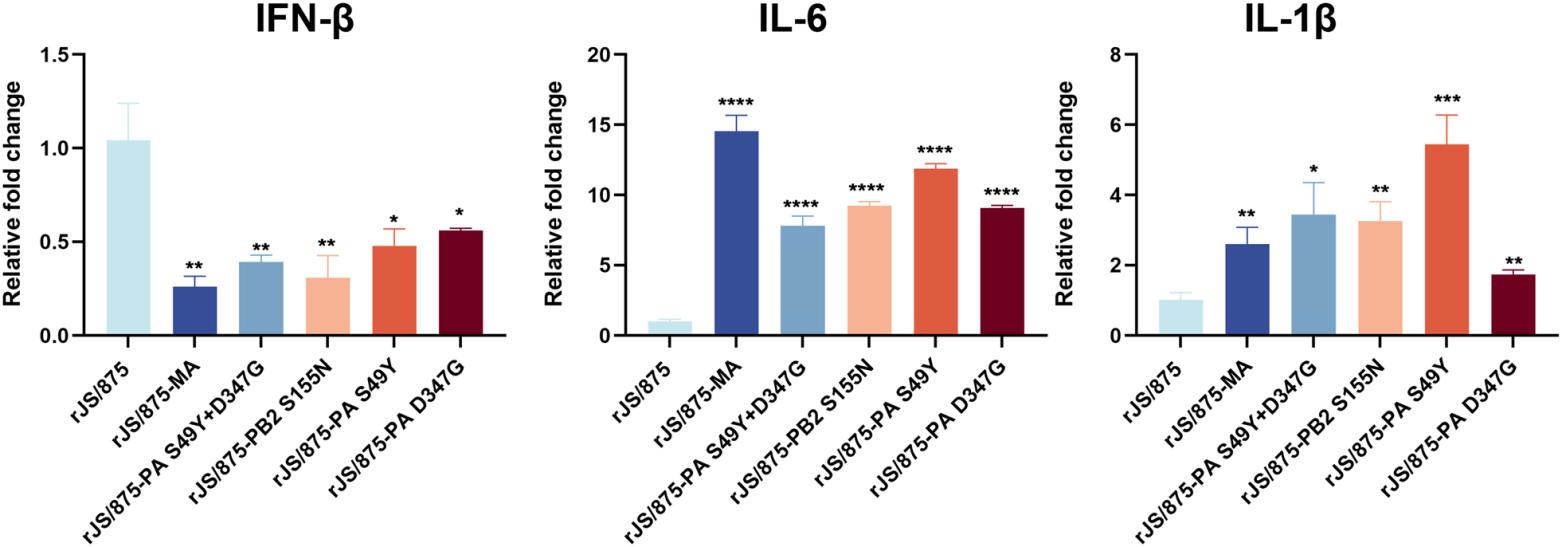
**The relative expression levels of cytokines in mouse lung tissue infected with recombinant viruses carrying the PB2 S155N and PA S49Y and D347G mutations.** The levels of cytokines in the infected mouse lung tissues are shown as the fold-change higher than the wild-type-infected group on day 3 post-infection. The data presented are the mean ± SD of three independent experiments. *P* values were calculated by using the Student’s *t* test, *P < 0.05, **P < 0.01, ***P < 0.001, ****P < 0.0001.

To evaluate the effect of these three key amino acid mutations on the production of cytokines in mouse lung tissue, we compared the induction of cytokines in the lungs of mice infected with wild-type rJS/875 and the different mutant viruses. As shown in Figure 7, the introduction of mouse-adapted mutations decreased IFN-β production compared to that in rJS/875, with the most prominent effect observed with virus rJS/875-MA. Compared to the lungs of the mice infected with the wild-type rJS/875 virus, exposure to the rJS/875-MA, rJS/875-PA S49Y+D347G, rJS/875-PB2 S155N, rJS/875-PA S49Y and rJS/875-PA D347G viruses led to significantly higher levels of the cytokines IL-6 and IL-1β on day 3 post-infection (Figure 7). The upregulated expression of IL-6 and IL-1β, the main cytokines of the inflammatory response, predicted an increased inflammatory response in the body, which is consistent with the increased inflammatory cytokine infiltration observed in the lung tissue of mutant virus-infected mice. The results indicated that these three mammalian adapted H9N2 viruses increased cytokine induction but decreased IFN-β production in mouse lungs.

### The PB2 S155N and PA S49Y and D347G mutations enhance polymerase activity in human cells but not in DF-1 cells

**Figure 8:**
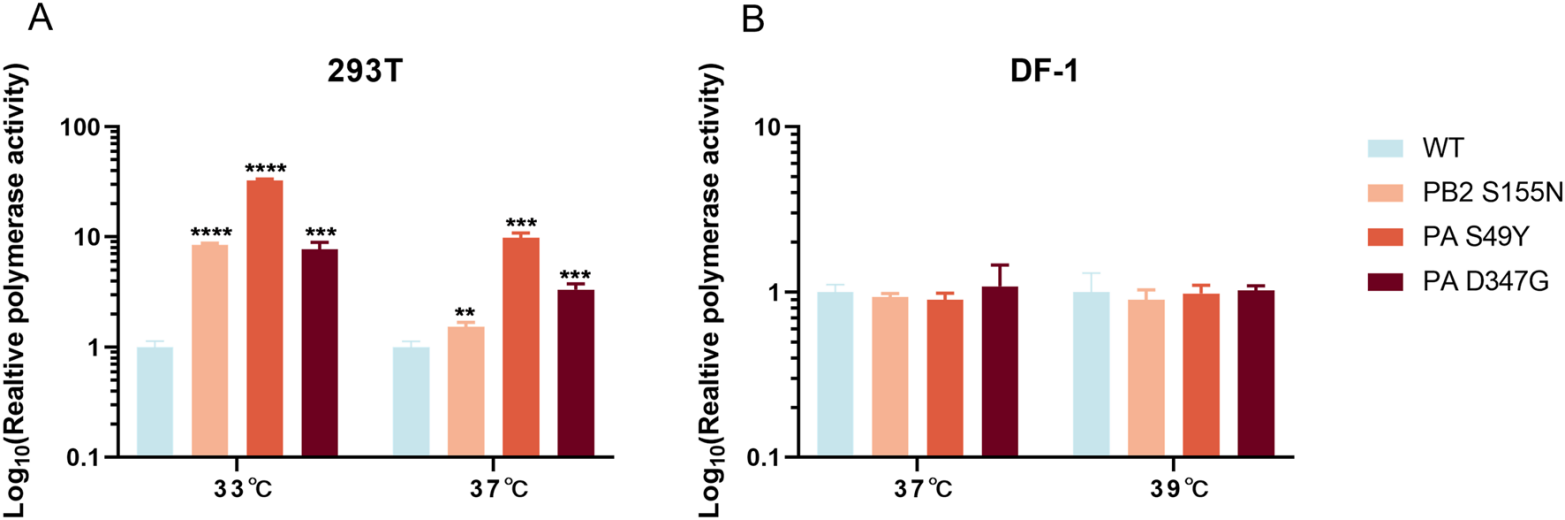
**Effects of the PB2 S155N and PA S49Y and D347G mutations on viral polymerase activity in 293t and DF-1 cells.** 293T cells (**A**) or DF-1 cells (**B**) were transfected with rJS/875 wild-type or mutant RNP complexes composed of PB2 WT or PB2 S155, PB1 WT, PA WT or PA S49Y, PA D347G and NP WT protein expression plasmids, together with the firefly luciferase reporter and a Renilla luciferase reporter (internal control). Luciferase activity was measured at 48 h post-transfection at 33 ℃ or 37 ℃. The data presented are the mean ± SD of three independent experiments. *P* values were calculated by using the Student’s *t* test, *P < 0.05, **P < 0.01, ***P < 0.001, ****P < 0.0001.

To evaluate the effect of the single amino acid mutations of these three key mammalian-adapted markers on host polymerase activity, a mini-genome assay was performed in mammalian (human 293T cells) and chicken model cells (embryo fibroblast DF-1 cells). The results showed that the single amino acid mutations PB2 S155N and PA S49Y and PA D347G led to a significant increase in polymerase activity in 293T cells at 33 and 37 °C (*P* ≤ 0.001) compared to the polymerase complex of wild-type virus. Moreover, the polymerase activity induced by the PA S49Y mutation was the strongest, which was 32.5 and 9.9 times higher than that of the wild-type virus at 33 and 37 °C, respectively (Figure 8A). In contrast to the results in human 293T cells, the effect of these three mutations of H9N2 viruses in avian hosts was measured in chicken embryo fibroblast DF-1 cells. The results showed that RNP polymerase activity remained unchanged with the single amino acid mutations in DF-1 cells at both temperatures (Figure 8B). These results demonstrated that PB2 S155N, PA S49Y and PA D347G were species-specific regulators that significantly increased the viral polymerase activity of avian H9N2 virus in 293T cells but not in DF-1 cells.

### The PB2 S155N and PA S49Y and D347G mutations also increase the polymerase activity of viruses with other backbones

**Figure 9:**
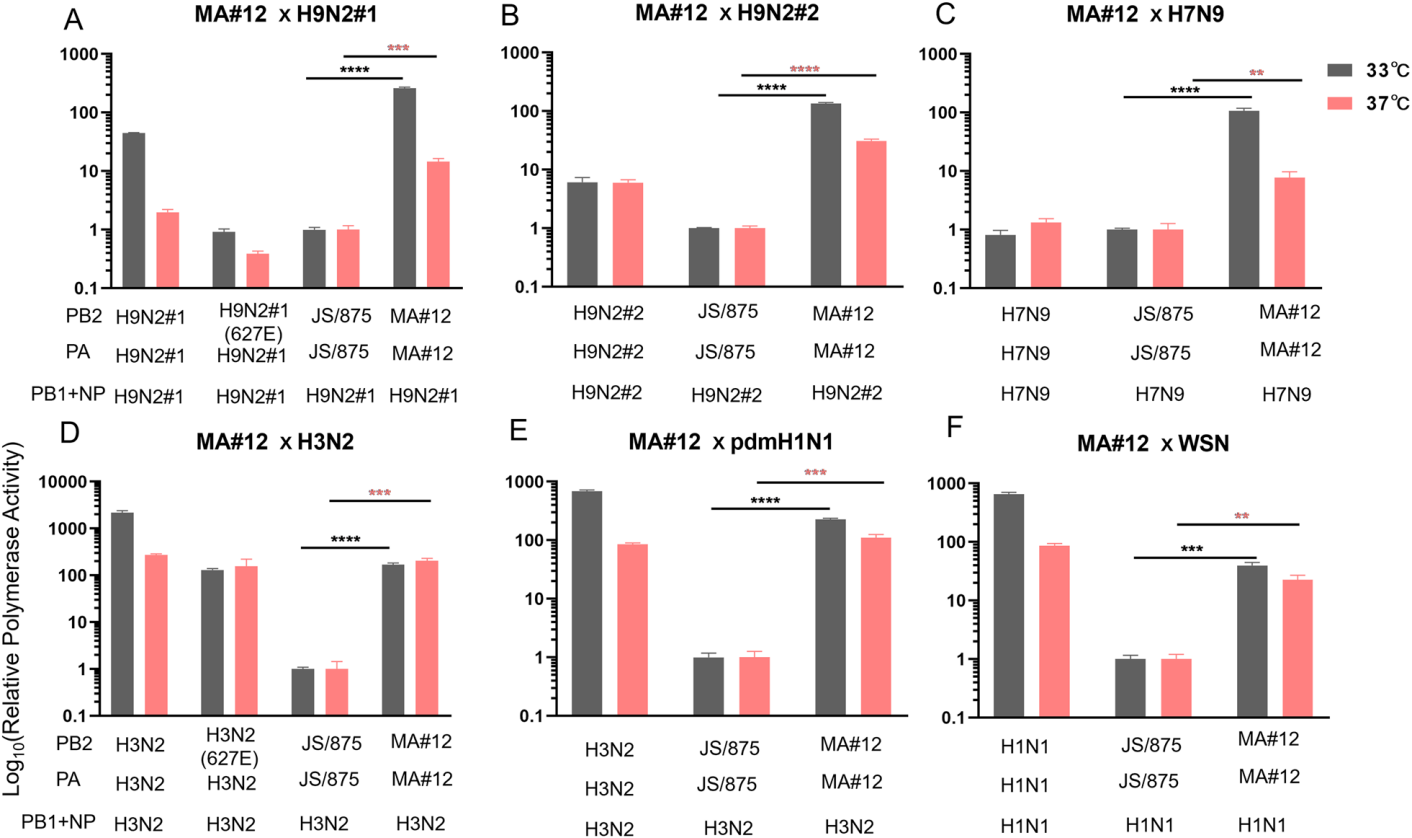
**The polymerase activities of wild-type viruses, re-assortant viruses and mouse-adapted viruses at 33 and 37 °C.** 293T cells were transfected with plasmids expressing PB1, the NP of 99-H9N2 (**A**), SD6-H9N2 (**B**), PG/S1421-H7N9 (**C**), HK-H1N1 (**D**), CAL04-H1N1 (**E**) and WSN-H1N1 (**F**) and wild-type or MA#12 PB2 and PA proteins at 33 ℃ and 37 ℃, and the polymerase activities were determined. During transfection, WSN NA-firefly and pRL-TK plasmids were transfected simultaneously. The data presented are the mean ± SD of three independent experiments. *P* values were calculated by using the Student’s *t* test, *P < 0.05, **P < 0.01, ***P < 0.001, ****P < 0.0001.

To determine whether the three key amino acid sites have a role only in JS875, their effects were determined in viruses with different viral backbones. A few representative viruses were selected, A/chicken/Jiangsu/99/2017(99-H9N2), A/chicken/Shandong/6/1996(SD6-H9N2), A/pigeon/Shanghai/S1421/2013(PG/S1421-H7N9), A/Hongkong/1/1968(HK-H3N2), A/California/04/2009(CAL04-H1N1) and A/WSN/1933(H1N1). Their PB2 and PA proteins were replaced with the wild-type and mutant PB2 and PA of rJS/875, respectively, and the polymerase activity of the reconstituted RNP complexes was assayed in 293T cells by using a luciferase minigenome assay at 33 and 37 °C. As expected, following the replacement of the PB2 and PA genes from MA#12, the polymerase activity of 99-H9N2, SD6-H9N2, PG/S1421-H7N9, HK-H1N1, CAL04-H1N1 and WSN-H1N1 was 261.2, 134.0, 107.1, 168.5, 228.6 and 39.8 times higher than that of wild-type JS/875-PB2/PA at 33 °C, respectively. At 37 °C, the polymerase activity of the substitution of the MA#12 PB2 and PA genes was amplified by 14.5, 31.1, 7.7, 204.8, 110.3 and 22.6 times that of wild-type JS/875-PB2/PA, respectively (Figures 9A–F). The results suggest that these three key amino acid mutations are not only relevant to the JS/875 virus but also have a major impact on the polymerase activity of various virus subtypes.

### The PB2 S155N and PA S49Y and D347G mutations conferred a growth advantage in mice

**Figure 10:**
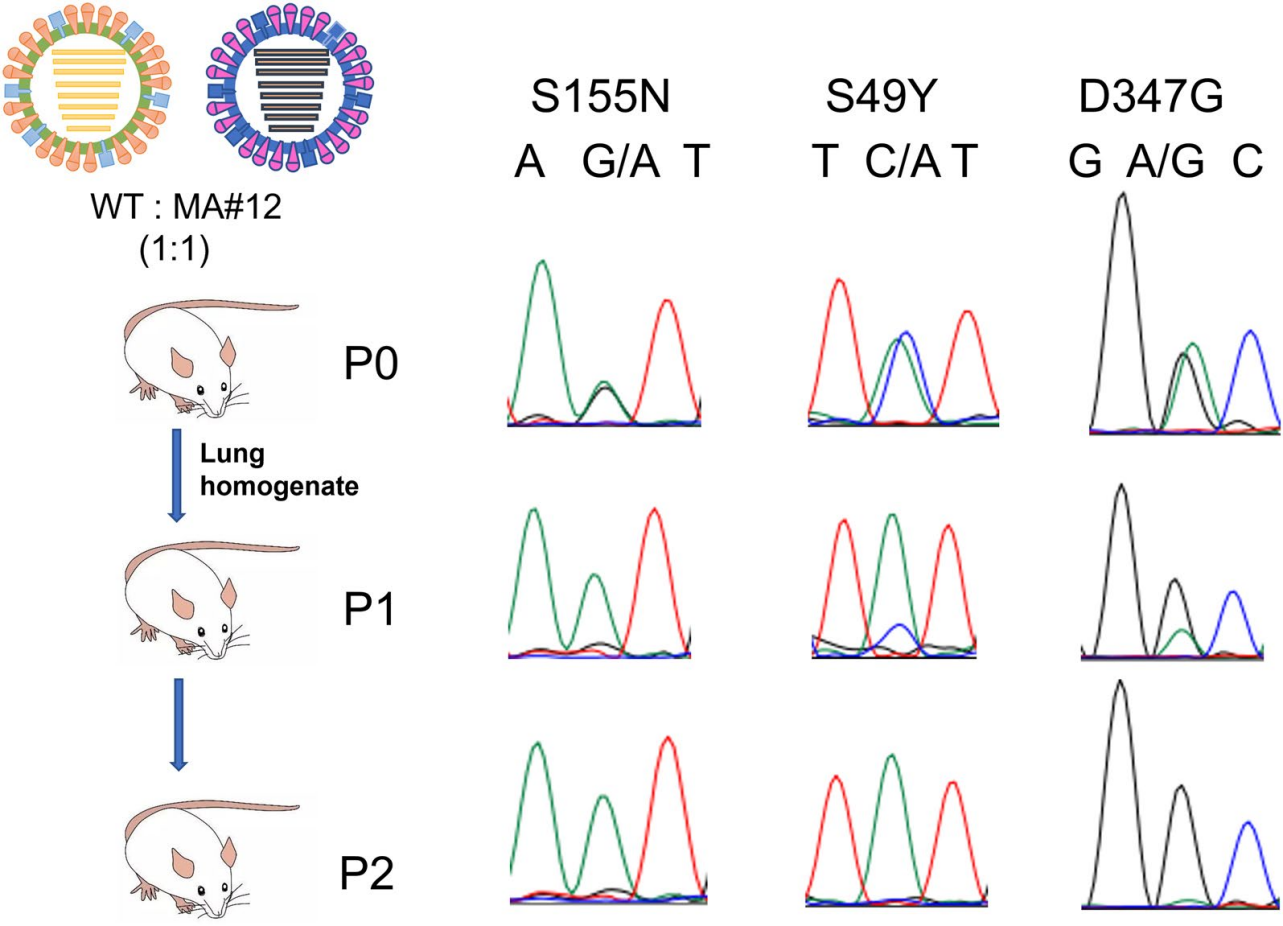
**The growth rate of rJS/875 and rJS/875-MA in mixed infection.** The rJS/875 and rJS/875-MA (MA#12) viruses were mixed at a 1:1 ratio (P0) to infect mice (*n* = 3), and on day 3 after infection, the mice were euthanized, and the supernatant of mouse lung tissue was collected (P1) and used to infect the next three mice (P2). P0, P1 and P2 viral fluids were collected, RNA extraction, fragment amplification and sequencing were performed, and the peaks of the PB2 S155N and PA S49Y and D347G positions were observed using Chromas software.

To further assess whether these three key amino acid mutations could become dominant and be preserved during natural infection, a mixed infection with two viruses in mice was simulated. The rJS/875 and rJS/875-MA(MA#12) viruses were diluted to the same virulence and then mixed in equal volumes (P0) to infect female BALB/c mice. On day 3 after infection, mice were euthanized, their lung tissues were dissected, homogenized in PBS, and centrifuged, and the supernatant was collected (P1). Subsequently, mice infected with P1 and P2 were assessed. The PB2 and PA gene sequences of the P0, P1 and P2 viruses were sequenced, and the nucleotide peaks of these three mutation sites were analysed using Chromas software. The sequencing of the P0 virus, created from a combination of the rJS/875 and rJS/875-MA viruses in a 1:1 ratio, displayed both sets of peaks in the results. Upon reaching the P1 generation, the mice had already accumulated a significantly greater number of mutated genes compared to unmutated genes. When passed to the P2 generation, the unmutated genes were largely eliminated, and the genes with mutations were overwhelmingly dominant (Figure 10). The results proved that gaining the PB2 155 N and PA 49Y and 347G mutations provided an additional growth advantage to the H9N2 virus. These results suggest that these three key amino acid mutations tend to become increasingly predominant in mammals in a natural state.

## Discussion

Host barriers usually impede the spread of viruses between species, yet when these interspecies barriers are breached, avian influenza viruses can infect mammals and humans [26]. Key amino acid mutations in the ribonucleoprotein (RNP) are one of the major adaptative mechanisms that enable avian influenza viruses to cross species boundaries and infect humans or other mammals [27]. H9N2 avian influenza viruses, which have been known to infect humans and have undergone genetic mutations, should not be overlooked as potential pandemic threats [28]. PB2 627K and 701N easily appear during viral evolution for most avian influenza viruses [19–21, 29], which may prevent the identification of some other important host-determining sites. Our only purpose was to identify other mammalian adaptative molecular markers in the polymerase and NP genes that were masked by the function of PB2 627K and 701N.

The bases of nucleotides are divided into the following two categories according to their ring structure characteristics: purines, including adenine A and guanine G (two-ringed structure), and pyrimidines, including cytosine C and thymine T (one ring structure). If a DNA base substitution maintains the same number of rings, it is called a transition; for example, adenine A is replaced by guanine G, or cytosine C is replaced by thymine T. If the number of rings changes, it is called transversion; for instance, adenine A is replaced by cytosine C, or thymine T is replaced by guanine G. There is a general consensus that transition mutations occur much more frequently than transversion mutations during the evolutionary process [25]. H9N2 avian influenza viruses are not natural pathogens of mice, and most of them are not pathogenic to mice. Serial passages of most avian influenza viruses in mice enable the virus to rapidly acquire PB2 E627K or D701N mutations that increase viral virulence, which obscures the function of other relatively important mammalian adaptive residues [29]. The H9N2 vaccine-like strain rJS/875 was passaged in mice in this study, and the PB2 627K mutation appeared during the third passage (data not shown). A previous report showed that recombinant PR8 virus derived from MDCK cells containing 627S and 627 L mutations replicated poorly in MDCK cells and exhibited moderate pathogenicity in mice [30]. In the present study, PB2 627E and 701D of the H9N2 virus were changed to serine (S) and leucine (L), respectively, by mutating three and two bases of nucleotides, respectively, through transversion (Figure 1), thereby reducing the possibility of the rapid acquisition of the E627K or D701N mutations during viral adaptation in mice. A highly pathogenic variant, rJS/875-MA, was obtained after twelve serial passages. We demonstrated that the PB2 S155N and PA S49Y and D347G amino acid mutations selected for upon mouse adaptation were key virulence determinants responsible for the observed increased in pathogenicity. These three amino acid mutations improved the growth characteristics in mammalian cells, promoted viral genome transcription and replication, increased viral polymerase activity and rendered the viruses lethal in mice. All three residues were mutated simultaneously and significantly increased the polymerase activity and replication of rJS/875, which supports the theory that staggering viral polymerase activity and viral replication contribute to the adaptation and high pathogenicity of H9N2 avian influenza viruses in mice [31, 32]. The functional regions of these sites were also analysed, and it was found that PB2 S155N is situated in the region of interaction with NP, which may affect the formation of the polymerase complex [33]. A previous study showed that the IAV PA protein can inhibit the production of IFN-β and interferon-stimulated genes induced by Sendai virus through interferon regulatory factor 3 (IRF3), and the N-terminal endonuclease activity of PA is responsible for its interaction with IRF3 and inhibition of the IFN-β signalling pathway [34]. The PA-S49Y mutation is in the PA-N domain of the polymerase complex, which could regulate the nucleic acid endonuclease activity of the PA protein [35]. Our hypothesis is that the PA-S49Y mutation may promote viral replication by suppressing the expression of IFN-β through interfering with the interaction of the PA protein and IRF3. The PA-D347G mutation is in the PA-C domain of the polymerase complex [36]. Previous studies found that the amino acid at position 347 of PA is located at the edge of an α-helix, the same as the amino acid at 336, which has been proven to affect viral polymerase activity and the pathogenicity of recombinant influenza viruses [37]. Moreover, our research also confirmed that the JS/875 PB2 and PA segments, which contain the PB2 S155N and PA S49Y and D347G mutations, were sufficient to increase the polymerase activity of complexes of several other subtypes of viruses, such as H7N9, H3N2 and H1N1 influenza viruses. We speculated that avian-derived PA and PB2, perhaps harbouring some host-adapted mutations, could drastically change the characteristics of the virus. This theory may be supported by the fact that the 2009 pandemic virus harboured two segments of avian origin, PA and PB2, with the other segments originating from swine influenza viruses or the human H3N2 influenza virus [38].

Host-adaptative substitutions in the ribonucleoprotein complex are critical for the replication of avian influenza viruses in mammalian hosts. A previous report showed that the D347N mutation was vital for the adaptation of H9N2 avian influenza viruses [39]. Subsequent research confirmed that PA-A343S/D347E mutations were critical for increasing the polymerase activity and mouse virulence of H5N1 viruses [36]. In addition, another research study found that the PA D347G mutation was vital for the host adaptation and viral virulence of the H7N9 virus [40]. In this study, we also observed the PA D347G mutation in mouse-adapted viruses and confirmed that it was essential for the mammalian host adaptation of the H9N2 influenza virus subtype. It is noteworthy that amino acid 347 of PA is significant in various avian influenza viruses despite their different mutant forms, and the specific mechanism by which this mutation affects virulence will need to be studied in the future. Several key mammalian adaptative molecular markers were obtained in multiple H9N2 mouse-adapted variants in this study. Song et al. reported that polymerase complexes with the PB2 K526R mutation derived from H7N9, H5N1 or H3N2 viruses could increase the viral polymerase activity, viral replication ability and mouse pathogenicity of the H7N9 subtype virus [41]. The PA E684G mutation has been proven to increase the viral replication efficiency of reassortant PR8 virus in ECEs, MDCK cells and viral pathogenicity in mice [42]; these two amino acid mutations were also observed in this study. PA T97I is a typical mouse-adapted mutation in multiple subtypes of influenza viruses [32, 43]. We also identified this mutation in several mouse-adapted viruses from two mice. Surprisingly, nine of the twelve adaptive variants obtained the PA T97I mutation, which was isolated from two mice. None of the mouse-adapted viruses isolated from the third mouse (MA#10 to MA#12) acquired the PA T97I mutation but they instead obtained PA S49Y and D347G mutations, suggesting that these two substitutions were capable of compensating for the absence of PA T97I to allow the cross-host transmission of avian H9N2 influenza viruses. Furthermore, sequence analysis in the present study showed that no adapted viruses obtained the Q226L and G228S mutations on the HA gene, which is vital for making the viruses lose affinity for α-2,3-linked sialic acids and increase affinity for human α-2,6-linked sialic acids [44]. This may be because the avian H9N2 influenza viruses isolated in recent years have dual receptor-binding properties, and most of them have higher binding capacity to human receptors than to avian receptors, indicating that these strains have largely adapted HA genes and therefore have not developed the Q226L and G228S mutations [45, 46]. In addition, there were also some novel mutations mapped to ribonucleoprotein as follows: PB1 V418I and L695I; PA I178M, M374T and V450A; and NP V183I, which contributed to the cross-host infection of avian H9N2 influenza viruses. However, their functions are not fully understood. Previous studies have proposed that mutations of the polymerase complex are essential for effective host adaptation [47, 48]; thus, it is reasonable to conclude that these mutations may be needed for communication between the polymerase and host factors, which could result in improved replication and transcription of the adapted virus in mammalian hosts.

We also investigated the role of viral RNP polymerase in viral transcription (mRNA) and genomic replication (vRNA) according to previous reports [41, 49]. This is currently recognized as a convenient and inexpensive method. The results showed that mutant viruses were able to boost viral mRNA and vRNA expression to some extent. However, the degree of promotion was not always in line with the virus’s capacity to replicate in A549 cells. We hypothesize that this may be due to the low specificity of the conventional real-time RT‒PCR method. Because viral cRNA and mRNA sequences are almost identical except for the cap structure of the 5′ end and the poly A tail of the 3′ end, some overlap is inevitable between the primers used to synthesize cDNAs for cRNA and mRNA. In addition, Kawakami et al. reported that the amplification of vRNA was observed not only in the progress of using vRNA-specific primers but also when no primer was added to the reverse transcription reaction [50]. They proposed a new method for distinguishing influenza vRNA, cRNA and mRNA, called hot-start reverse transcription with a tagged primer. By using primers with specific tags for reverse transcription and PCR, nonspecific amplification was reduced, which demonstrated the superiority of this method. We will refer to this new method for more precise conclusions when conducting relevant experiments in the future.

The low polymerase activity of avian influenza viruses in mammalian cells is one of the major factors limiting virus cross-host infection [17]. In this study, a minigenome assay confirmed that all mouse-adapted mutations enhanced the polymerase activity of the virus in 293T cells. The most dramatic differences were observed at 33 °C (which is close to the temperature of the human upper respiratory tract), while at 37 °C, these differences were far less pronounced. We observed similar results in other studies; for example, the difference in polymerase activity between PB2 627E and 627 K in 293T cells was greater at 33 °C than at 37 °C [51, 52]. This may be because 39 °C is more conducive to avian-derived virus replication and 33 °C is more suitable for human-derived virus replication. However, 37 °C is suitable for both avian and human-derived viruses. Therefore, viral polymerase activity at 37 °C was far lower because both the avian-derived virus rJS/875 and mouse-adapted viruses replicated well at this temperature.

In this study, three important mouse-adaptive molecular markers, PB2 S155N and PA S49Y and D347G, were identified. However, due to the restriction of strain type, it is possible that the mutations we identified are strain specific. The cross-host infection of avian influenza viruses is often determined by a combination of multiple gene and multiple amino acid mutations, and some mutations are strain specific. It is worth further investigating whether the three key amino acid mutations identified in this study have a role in the cross-host infection of other avian influenza viruses, such as the H5, H7, and H10 subtypes.

Overall, we demonstrated here that three substitutions, PB2 S155N and PA S49Y and D347G, contribute to the high virulence of rJS/875-MA in mice in the absence of PB2 627K or 701N, resulting in increased polymerase activity, viral transcription, genomic replication, and virus yields in mammalian cells, induced proinflammatory cytokine responses in the lungs of virus-infected mice and improved growth rates. Our results suggest that the detection of these three amino acids in upcoming H9N2 influenza viruses may signal an increased risk for severe infection in mammals, which has important guiding significance for the prevention of future pandemics.

### Supplementary Information

**Additional file 1.** **Sequences of primers used in this study.**
