# Supplementary material for: Exploring the alternative virulence determinants PB2 S155N and PA S49Y/D347G that promote mammalian adaptation of the H9N2 avian influenza virus in mice
Source: Vet Res. 2023 Oct 19;54:97. doi: 10.1186/s13567-023-01221-6 (PMC10588254; doi:10.1186/s13567-023-01221-6)
Supplement: Supplementary file 1 — Additional file 1. Sequences of primers used in this study. [file 13567_2023_1221_MOESM1_ESM.docx]

**Additional file 1 Primer sequences used in this study.**

| Primer name | Sequences |
| --- | --- |
| NP vRNA primer | 5’-AGCAAAAGCAGGGTAGATAATCACT-3’ |
| NP-F | 5’-GGATGTGCTCTCTGATGCAA-3’ |
| NP-R | 5’-CTTTGTGCTGCTGTTTGGAA-3’ |
| Human-GAPDH F | 5’-ACAACTTTGGTATCGTGGAAGG-3’ |
| Human-GAPDH R | 5’-GCCATCACGCCACAGTTTC-3’ |
| Mus- IFN-β F | 5ˊ-TGGGTGGAATGAGACTATTGTTG-3ˊ |
| Mus- IFN-β R | 5ˊ-CTCCCACGTCAATCTTTCCTC-3ˊ |
| Mus-IL-1β F | 5ˊ-GAAATGCCACCTTTTGACAGTG-3ˊ |
| Mus-IL-1β R | 5ˊ-TGGATGCTCTCATCAGGACAG-3ˊ |
| Mus IL-6 F | 5ˊ-CTGCAAGAGACTTCCATCCAG-3ˊ |
| Mus IL-6 R | 5ˊ-AGTGGTATAGACAGGTCTGTTGG-3ˊ |
| Mus GAPDH F | 5ˊ-TGACCTCAACTACATGGTCTACA-3ˊ |
| Mus GAPDH R | 5ˊ-CTTCCCATTCTCGGCCTTG-3ˊ |
